# A Hand Book of Therapeutics

**Published:** 1873-02

**Authors:** 


*A Handbook of Therapeutics.* By Sidney Ringer, M. D. Third Edition. New York: Wm. Wood & Co., 1872. Buffalo, H. H. Otis.

Not quite one year ago we had occasion to express our appreciation of this work in the pages of this journal; since that time the author has made valuable improvements and additions, and the call for a new edition in so short a time is evidence that it is highly appreciated by the professional public.

The book, as now presented, is admirably arranged, and the contents evince a wide range of study and thought by the author.

